# Qualitative content analysis as a research method to investigate hazards information in school textbooks

**DOI:** 10.1016/j.mex.2021.101559

**Published:** 2021-10-29

**Authors:** Hamed Seddighi, Sepideh Yousefzadeh, Mónica López López

**Affiliations:** a Governance and Innovation Department, Campus Fryslân, University of Groningen, Leeuwarden, the Netherlands; b Campus Fryslân, University of Groningen, Leeuwarden, the Netherlands; c Faculty of Behavioural and Social Sciences, University of Groningen, Groningen, the Netherlands

**Keywords:** Disaster education, Curriculum, Climate change, Content analysis

## Abstract

This study explains how qualitative content analysis was applied to investigate natural hazards in textbooks for children with intellectual disabilities in Iran. Qualitative content analysis of textbooks is one of the ways for understanding the priorities of Iranian education system with regard to natural hazards. Data samples included whole textbooks for children with intellectual disabilities in all grades during school year 2020-2021 in Iran. Data were collected by transferring textbooks to MAXQDA 2018 software and coding themes with the software. A narrative format was used for analyzing qualitative data. Examples were presented along with tables and quotations in the study. Peer checking and expert check were employed to ensure trustworthiness of the study. The above research design showed the strengths and weaknesses of information provided in Iranian textbooks on natural hazards and disasters management. Iran is a disaster-prone country and various natural hazards happen in Iran every year including earthquake, flood, drought, and extreme weather. Children are a vulnerable population in disasters. One of the most important issues for children's health is disaster preparedness. Intersection of age with other social determinants such as disability, gender, and ethnicity can increase vulnerability.

This method was recommended to researchers investigating representation of natural hazards in textbooks of children in different countries, grades, and textbooks. Furthermore, it is possible to use this method for a comparative analysis of information in two or more countries, or different school years in a country.

• Explicit and implicit information on natural hazards in textbooks could be investigated with qualitative content analysis.
• This method could facilitate cross-country comparisons by providing a framework to investigate the content of textbooks for children without disabilities and/or with disabilities.
• It is a valuable method for evaluation of disaster programs for children in local, national, and global levels.

## Graphical abstract

**Figure:**
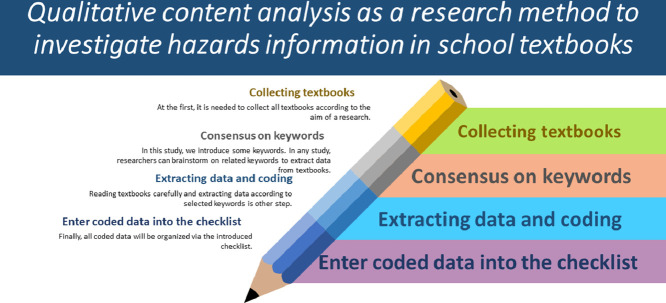

**Specifications table**

**Table:** 

Subject Area	Environmental Science
More specific subject area	*Children study, Gender study, Disaster study*
Method name	*Qualitative content analysis*
Name and reference of original method	*Drisko, J.W. and T. Maschi, Content analysis. 2015: Pocket Guides to Social Work R.*
Resource availability	*N.A.*

## Background

Textbooks are one of the most important educational materials for both teachers and students in schools [1]. To understand the priorities, values and ideologies of a country, investigating textbooks is an efficient way [2]. One of the most important issues of recent decades is climate change, which has increased natural hazards [3]. Increasing natural hazards, urbanization, as well as social determinants of vulnerabilities have led to more people being affected by disasters [4]. Children are one of the most vulnerable groups in disasters, particularly children with intellectual disabilities. Iran is prone to various natural hazards such as earthquakes, floods, and droughts [5]. In this study, it was examined the textbooks of children with intellectual disabilities using qualitative content analysis as a method to find out to what extent the natural hazards are reflected in the textbooks of these children.

## Method details

Qualitative content analysis includes a set of techniques for systematic analysis of texts and media. This method helps to interpret the hidden meanings in texts and images [6]. Qualitative content analysis goes beyond word counting and quantitative content analysis and reveals the meanings implicit in words.

The following research questions (RQ's) were designed to achieve the purpose of this study:

• Have all textbooks addressed the issue of natural disasters and how much have they covered the issue?
• How do all students at all levels get information about natural hazards through textbooks?
• Do textbooks address disaster management and the causes and effects of natural hazards, and if yes, how??
• Are local hazards mentioned in textbooks, and if yes how?
• How are gender and diversity portrayed in the content of textbooks on natural hazards?

## Target Audiences

Target audience for this qualitative content analysis falls into three groups including policy makers, researchers, and fieldworkers.

*International researchers:* explain that the study adds to the knowledge base on the topic, but it might also serve as an inspiration on how to use this method, to explore the issue in other countries.

*Policy makers and professionals in the field in the country:* attention to the topic, learning how the information is transmitted and the knowledge gaps that should be tackled.

*Fieldworkers at an international level, or particularly working in disaster prone areas:* This group should be aware of the extent to which the information is transmitted through textbooks to children with ID. Once it is clarified, they can take action to improve the available trainings and to fill or remove the gaps.

## Sampling

In this study, whole textbooks of the education system in Iran for children with ID were studies (total population sampling). Samples for qualitative content analysis should be appropriate to RQ's, and information has to be rich and adequate enough to survey RQ's. As a result, the first step included the collection of school textbooks for children with ID in Iran. The Iranian Education Ministry had uploaded all textbooks to a website [7]. Thus, 130 textbooks were downloaded those used in special schools for children with ID in the 2020-2021 educational year. Primary school relevant textbooks were categorized into special textbooks for children with ID, children with ID & visual impairment, and students with autism. Among whole 130 textbooks for children with ID in Iran, 41 textbooks are provided in secondary schools.

## Data collection

**Fig. 1:**
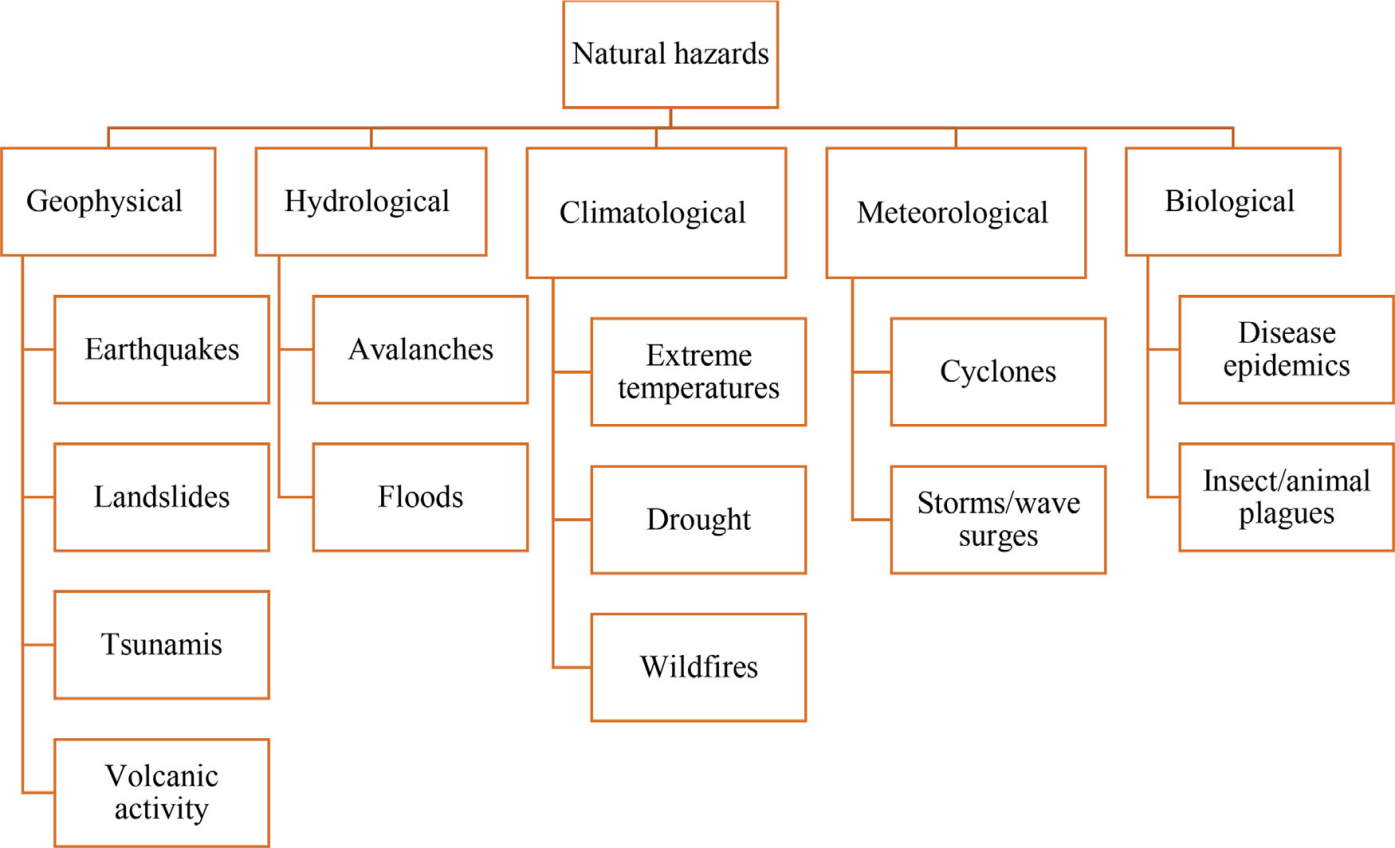
Example of keywords of natural hazards.

**Fig. 2:**
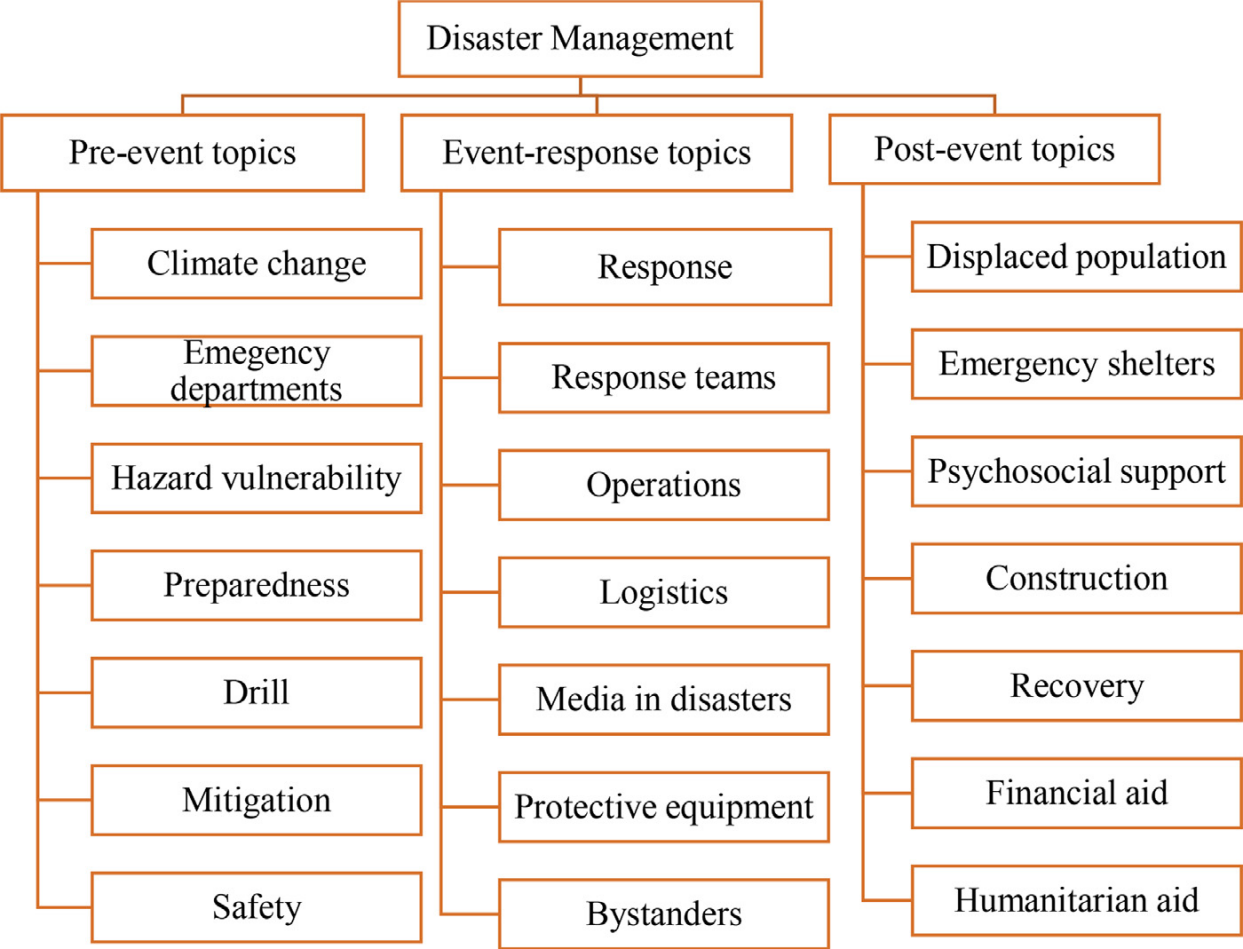
Example of keywords of disaster management.

All electronic versions of textbooks for students with ID were entered into MAXQDA 2018 software [8]. Texts and images and other content related to natural hazards were investigated according to keywords. The keywords related to natural hazards were selected according to the literature review, authors’ brainstorming, and coding during data collection. In addition to using known keywords, the authors generated code while collecting data. These keywords were categorized into two topics including natural hazards and disaster management. The keywords are shown in Figs. 1 and 2. During the process, feedback from an expert in disaster studies, an expert in gender studies, and an expert of childhood studies were collected in data collection process.

The extracted data from textbooks and, including title of textbook, grade, related text, related image, number of pages dedicated to the related topic, total pages of textbook, characters' gender and diversity, and role of characters. The ratio of relevant pages to the total number of pages in the book may contribute to the analysis of findings. It also can be useful to compare the volume devoted to natural hazards in different years in the same textbook as well as in comparisons made between different countries. Data extraction form is shown in Table 1.

**Table 1:** Data extraction form for collecting data from textbooks.

Title of Textbooks	Grade	Content related to natural hazards	Number of pages devoted to the related topic	Total pages of textbooks	Characters’ gender	Diversity of characters in the topic (i.e. diversity, ethnicity)	Role of characters (i.e. aid workers, rescuers, firefighters)	Type of hazard discussed in the topic (i.e. flood)
								

## Data analysis

A narrative method was employed for data analysis. Themes and sub-themes as were used section headings in the study. Each theme or subtheme was briefly interpreted. Quotations, and tables were used to present examples. There are several benefits of presenting examples in the narrative format of data analysis: it highlights categorization, clarifies how the research questions are answered, and shows how the themes or categories were developed [6]. In addition, readers can use tables to track data and apply them to decide about themes and subthemes. Also, researchers can use the examples presented within tables in their future studies. Table 2 was applied to show some examples of representation of natural hazards in the school textbooks. In addition, Table 3 represents some examples about gender and diversity in the content related to natural hazards in textbooks of students with ID.

**Table 2:** Findings from the textbooks for children with ID.

No.	Grade	Textbooks	Content	Page(s)	Total pages

**Table 3:** Representation of gender and diversity in the content related to natural hazards.

No.	Grade	Textbooks	Character (gender/disability/ethnicity)	Role of character	Emergency topic
					

## Trustworthiness

Peer checking and expert check were used to enhance trustworthiness of the study. Coded data were reviewed by other members of the research team who specialized in child studies and disaster studies. In addition, an expert in disaster studies checked the coded data and improved open coding. Coded data was shared with experts. After reviewing the codes by the experts, the texts and images of all textbooks were re-reviewed to ensure that the researchers did not miss a point in the first review.

## Conclusions

Qualitative content analysis was a powerful tool for us to investigate textbooks to find representation of natural hazards. This analysis applied a narrative format for analyzing implicit and explicit content devoted to natural hazards. This approach reveals implicit information hidden in texts and images. This research method was recommended for investigating representation of natural hazards in textbooks of children in different countries, grades, and textbooks. Furthermore, this approach can be used to perform comparative analyses dealing with information provided in textbooks in two or more countries, or different years in a country.

Policy makers, managers, relief and education organizations, the media and researchers all can use the results of textbook content analysis on natural hazards. Policy makers can identify policy silence and fill gaps. Managers and relief organizations can improve their trainings and remove the gaps in all cases, including the known training gaps through this method. The media can advocate children's preparedness for disasters. Researchers in different countries are also recommended to conduct similar research and produce scientific evidence in this regard.

## Declaration of Competing Interest

There was no conflict of interest among the authors. All authors read the final manuscript and approved of it.

The authors declare that they have no known competing financial interests or personal relationships that could have appeared to influence the work reported in this paper.
